# 

**Published:** 1916-03-25

**Authors:** 


### Annual Meetings of Special Interest.

Charing Cross Hospital, Agar Street, Strand, W.C.—Thursday, the 23rd inst., at 5 p.m., in the Council Room at the hospital, Her Royal Highness Princess Louise Duchess of Argyle, President of the hospital, has promised to attend. Colonel Verity, R.E., the Chairman of the hospital, will make a statement in regard to the hospital's position.

St. Peter's Hospital, Henrietta Street, Covent Garden, W.C.—Tuesday, March 28, at 5 p.m., at the hospital, F. A. Bevan, Esq., the treasurer, will preside.

The Infants' Hospital, Vincent Square, Westminster, S.W.—Wednesday, the 29th inst., at 4 p.m., at the hospital.

### Personalia.

Mr. F. R. Anderton has been elected chairman, and Mrs. Wilton Phipps vice-chairman, of the Public Health Committee of the London County Council.

Sir George Newman, chief medical officer of the Board of Education, has, owing to the pressure of the duties of that office, resigned his post as Lecturer on Public Health at St. Bartholomew's Hospital. The vacancy has been filled by Dr. R. A. Lyster, of The Castle, Winchester, the Lecturer on Forensic Medicine at the hospital.

At the annual meeting of subscribers to the Royal Alexandra Infirmary, Paisley, a tribute was paid to the splendid services rendered to the institution during a period of twenty-one years by Mr. John Abercrombie, the Hon. Secretary, who was re-elected to this post.

### HOSPITAL RESULTS IN 1915.

### Some Early Reports of the Voluntary Hospitals.

London Homœopathic Hospital.—The chair at the sixty-sixth annual general meeting was taken by the Earl of Donoughmore, a vice-president and treasurer of the hospital. For the fifth year in succession a large deficit in respect of ordinary income and expenditure was reported. Seventy-five beds reserved for sick and wounded have been in use by soldiers coming from the Dardanelles and elsewhere since October 1915.

Birmingham General Hospital.—This is one of the earliest complete reports published. One hundred beds are being reserved for the War Office. Forty extra beds over the normal number have been provided. From the financial statement it is gathered that it is the practice of the hospital to fund half the legacies received and to use the other half, if necessary, for current expenses; but the fortunes of the institution have varied so greatly in recent years that there was a surplus of nearly £8,000 in 1913, and deficits of £5,000 and £8,000 in 1914 and 1915 respectively. It is noticed that the number of out-patients treated has steadily fallen from 73,309 in 1911 to 50,879 in 1915, but no explanation of the fact is given in the annual report.

Northampton General Hospital.—This is one of the larger provincial hospitals not conforming with the usual custom of presenting accounts made up for the period January 1 to December 31, and at Northampton the financial twelve months closes on July 31. Fifty beds have been placed at the disposal of the County Red Cross Committee, and 715 wounded soldiers coming from abroad or belonging to the Home Forces were warded in twelve months. The income rose to £16,544, being £3,506 more than in the preceding twelve months, but there was a loss on the year's working of £910, leaving a total deficiency on revenue account of £3,618. Legacies, however (£1,251 in 1914-1915) were carried direct to an investment account.

Bristol General Hospital.—The advance copy of the committee's report records the completion of the new chapel, provided through the generosity of Mr. Fenwick Richards. The old chapel has been converted into a ward, and supplies much needed additional accommodation. Two wards, containing fifty beds, have been set apart for the use of wounded soldiers, and the number of cases admitted during the year was 138. The ordinary income shows an increase of about £2,500, and the annual subscriptions have increased by over £200. The expenditure, however, was materially heavier; the cost of provisions, to quote one item, being almost 50 per cent. higher than in 1914, and after all the legacies received (£4,647) had been used for maintenance purposes there was a substantial deficit on the year's working.

Queen's Hospital, Birmingham.—The seventy-fifth annual report records a substantial decrease in the number of out-patients treated—a feature noted in connection with several of the larger provincial hospitals. The wards, however, were used to their utmost capacity by military and civilian patients. The committee allude in feeling terms to the death of the matron, Miss Maude A. Buckingham, who had been granted leave of absence to take up the position of matron of the 2nd Birmingham War Hospital, where she died on December 2. An almoner's department has been started, and two members of the committee have undertaken the expenses of the new feature for the first twelve months. A deficit of £6,161 on the year's working has been made up by a transfer from legacy account.

Chelmsford and Essex Hospital and Dispensary.—A temporary Red Cross Hospital has been erected in the hospital grounds, and 257 soldiers have been received from the French front and the Dardanelles. The accounts of the hospital and dispensary show a surplus of £252 on the year's working. There is a very successful Linen League in connection with the institution.

Essex County Hospital, Colchester.—The women patients have been transferred to premises formerly occupied by a school in Wellesley Road, and the beds thus set free have been used for the wounded. There is a deficit of £992 on the year's accounts.

Blackburn and East Lancashire Royal Infirmary.—This is another example of an early published complete report presented in a well-arranged and convenient form. In Blackburn in-patients have increased in number and out-patients decreased in recent years. The ordinary expenditure has risen from £8,590 to £10,123, and the income from £9,006 to £9,428. Some soldiers were treated early in 1914, but in view of the long civilian waiting-list it has been arranged that the military authorities will not send soldiers until the list has been considerably reduced.

Royal Hospital for Diseases of the Chest, City Road, E.C.—The Lord Mayor (Colonel Sir Charles Wakefield) presided on the 16th inst. at the annual meeting. The entire hospital has since July last been devoted to the treatment of wounded soldiers. The hospital is not incorporated, but the bankers having declined to go further than the sum of £8,000 already advanced on loan, several members of the council consequently gave a guarantee which will facilitate the present financing of the institution. The out-patient department and tuberculosis dispensary are continued for the benefit of the poor of the district.

Gateshead Children's Hospital.—The annual report shows a deficiency on the year's working of £418. More work was done in 1915 than in 1914, and the average number of occupied beds rose from twenty-six to thirty.

Wolverhampton and Staffordshire General Hospital.—The number of out-patients fell from 18,963 to 15,856, while the wards were filled to about normal extent. The hospital must be congratulated on an excess of income over expenditure.

Hertford County Hospital.—Admiral Hastings, the Vice-Chairman of the Board, presided at the annual meeting on March 16. The accounts showed a balance in hand of £372. Amongst recent improvements at the hospital are an $x$-ray department and an electric lift.

The Cancer Hospital.—At the annual meeting, when Lord Northbrook, the Chairman of the Governors, presided, it was reported that the financial position continues to be satisfactory. During 1915, in the electrical and radio-therapeutic department, 135 military patients were treated, and 57 soldiers were admitted to the wards.

### KING EDWARD'S HOSPITAL FUND FOR LONDON.

We are asked to state that hospitals in the county of London or within nine miles of Charing Cross desiring to participate in the grants made by this Fund for the year 1916 must make application before the 31st instant to the Honorary Secretaries, 7 Walbrook, E.C. Applications will also be considered from convalescent homes which are situated within the above boundaries, or which, being situated outside, take a large proportion of patients from London. Applications will also be considered from sanatoria for consumption which take patients from London, or which are prepared to place beds at the disposal of the Fund for the use of patients from London hospitals.

### PASSING EVENTS AND TOPICS.

### Transport of Wounded by Sledges.

The Swiss colony in Paris has presented to the medical service of the French Army 100 sledges for the transportation of wounded soldiers in the Vosges.

### Tuberculous Soldiers in France.

Plans are being prepared for barracks to be erected on the available land of the Paris hospitals for the isolation and treatment of 2,500 discharged tuberculous soldiers.

### A Hospital's Centenary.

The Royal Waterloo Hospital for Children and Women reaches its centenary this year, and the Lord Mayor will preside at a public meeting to be held in the City on April 27 to celebrate the occasion.

### The Septic Slate.

The Board of Education are of opinion that the reintroduction of slates in schools is open to great objections on hygienic grounds. Moreover it is necessary, as with proper economy there is no risk of a shortage of paper.

### Medallions for Red Cross Helpers.

On Tuesday last week Princess Louis of Battenberg, accompanied by Admiral Prince Louis of Battenberg, presented medallions and certificates for home nursing and first aid gained by lady helpers at Northwood Red Cross Hospital.

### Motor Boats for Mesopotamia.

The motor-boat ambulances sent to Mesopotamia by the Red Cross authorities are doing excellent work. This it will now be possible to extend as a result of a gift of £9,000 from the fund, which owes its origin to Mr. Dennis Bayley.

### Royal Pavilion Hospital, Brighton.

During February more than ten thousand persons paid for admission to the Royal Pavilion Hospital at Brighton, which was formerly in the occupation of wounded Indian soldiers. The sum of £300 was realised and handed to the Mayor of Brighton for local charities.

### A Day Nursery for Birkenhead.

A day nursery has been opened at Birkenhead under the auspices of the Corporation, and it is the intention to establish similar institutions in the north and south ends of the town. The Corporation has also purchased a house for use as a child-welfare centre.

### Mortality in French Military Hospitals.

According to an official report the mortality rate in French military hospitals was 4.5 per cent. in 1914 and the beginning of 1915. Since then the rate has been greatly diminished, and out of 100 patients treated in military hospitals ninety-eight, on the average, are discharged recovered.

### The Royal Visit to Roehampton Hospital.

When the King and Queen paid a recent visit to the Queen Mary Auxiliary Hospital for Limbless Soldiers and Sailors at Roehampton their Majesties witnessed a remarkable demonstration in the Rowley Leg Department, and took particular interest in one of the soldiers with an artificial leg, who was able to run and jump quite freely.

### Scotland's Hospital Ship.

The Scottish Red Cross hospital ship *St. Margaret of Scotland* was recently opened by Lady Beatty, wife of Admiral Sir David Beatty. At the opening ceremony a telegram was received from the First Lord of the Admiralty, expressing his appreciation of the generous response on the part of Scotland to the appeal for subscriptions.

### An Old Harrovian's Valour.

The King of Italy has bestowed on Mr. G. M. Trevelyan the silver medal for military valour. Mr. Trevelyan took the first British ambulance unit to the Italian Front, and, according to the *Harrovian*, when a small cholera hospital at St. Florian, close to the Gorizia front, was being heavily bombarded by Austrian guns, he at once went to the spot and conveyed the inmates into safety, his motor being riddled with shrapnel.

### M.A.B. Expenditure.

The estimates of the Metropolitan Asylums Board for the half-year ending September 30, 1916, show a reduction of £32,750 compared with the corresponding period of 1915. At a meeting of the Board on March 4 it was stated that the decrease was mainly due to three hospitals having been taken over by the military authorities and to the exercise of the strictest economy in all the institutions under the Board's control.

### R.A.M.C. Comforts.

The committee of ladies which was formed early in the war for the purpose of collecting and forwarding comforts to the various medical units abroad has sent out altogether 2,040 bales, and of this number 607 bales, each weighing 11 lb., have been sent to prisoners of war, and 1,433 bales, averaging 50 lb. in weight, have been forwarded overseas. Lieutenant-Colonel F. W. H. Davis Harris, the treasurer of the fund, received up to the end of December £2,182 5s. 11d.

### A Hospital's Diamond Jubilee.

The Diamond Jubilee of the Great Northern Central Hospital, which occurs this year, would have been made the occasion of a festival had the times been propitious. The hospital has done its share of war work, and at the sixtieth annual meeting Mr. J. L. Smithett, who presided in the absence of Lord Northampton, who is at the Front, stated that last year 626 wounded soldiers had been treated in the wards, and in the out-patient department 1,200 soldiers had been treated.

### Royal Alexandra Infirmary, Paisley.

A sum of £2,250 has been added to the endowment fund of the Royal Alexandra Infirmary, Paisley, £1,250 being to endow a bed in memory of the late Mr. Robert Cochran. The infirmary has also received £1,000 legacy under the will of the late Mr. James H. Dunn, writer. The endowment fund now amounts to £89,445, exclusive of the due proportions of the residue of the estates of the late Mr. James Cowan, of Ross Hall, and the late Mr. James Jackson, and the entire residue of the estate of the late Mrs. Jessie Campbell or Barclay.

### Munition Workers' Generosity.

The workpeople at Elswick and Scotswood have presented an *x*-ray apparatus and sixteen new trolleys to the Northumberland War Hospital, Gosforth. The fund from which these appliances came owes its existence chiefly to Sister Beatrice Bartlett, who since the commencement of the war has paid some sixty visits to the Front, on each occasion taking material useful to the soldiers. The contributors to the fund comprise the workers in the ordnance, engine, and steel works, and since the war began £26,000 has been contributed.

### Blind Soldiers and Massage.

Eleven blind students have passed the examination held recently by the Incorporated Society of Trained Masseuses in either Part 1 or both parts of the examination. Of the five who entered for the whole examination, Mr. Percy Linney Way heads the list of over 200 candidates, while Mr. Percy Webb has obtained distinction. One of the blind candidates is a surgeon, and six are soldiers who have lost their sight on active service. The eleven blind candidates received instruction at the National Institute for the Blind, 224-6-8 Great Portland Street, W.

### A Russian Hospital Train.

A magnificently equipped Russian hospital train is described by a correspondent of the *Dundee Courier*. Most of the carriages, which number sixteen, are of great size, being fully 13 feet high. The wards and the doctors' and nurses' quarters are in the middle of the train. There is a post office, with three officials who sort the soldiers' letters picked up *en route*, a laundry, a dental surgery, a pharmacy, a tailor's workroom, and a variety of shops. In fact, this hospital train is like a little town on wheels. Cows and hens are kept on board, so that the train is independent of outside supplies of milk and eggs.

### Croydon Guild of Help.

The annual report of the Croydon Guild of Help shows that this organisation did much valuable work last year in supplying warm garments to consumptive patients, surgical appliances and extra nourishment to patients attending London hospitals, and in many other ways. The war brought its own special cases, a great number of which do not come within the scope of the Prince of Wales' Fund, and must therefore be helped by other bodies. The aggregate number of cases that the Guild was asked to help was rather less than in the previous year, but the work had to be done by a far smaller number of helpers than ever before.

### The G.W. Railway Ambulance Train.

The arrangements on the Great Western Railway's ambulance train, which was on view at Paddington Station last week, seemed excellent. We should, however, like to query one or two points. The sterilised-water supply for drinking purposes seemed inadequate; it was locked up, and the orderlies are not allowed a key. Four sisters are on this train, responsible for over 500 patients, some of whom may be, from the nature of their injuries, in urgent need of fluid. It would be useful if the authorities were to issue a leaflet to be given to the visitors by invitation to every new ambulance train, for without it it was not possible to see how the severe cases in the cots could be fed and watered. We should welcome information on these points and also to know why a Primus stove, which is not pleasant to use, has been selected, and not an electric heater in a train so liberally provided with electric fans! In each coach constituting a ward unit an electric heater should be provided for use in emergencies and for the preparation of hot applications for the relief of pain or for making bovril or a cup of tea.

### The Sale of Morphine to Soldiers.

In The Hospital of February 19 (p. 446), in a comment upon the practice of selling morphine to Army officers, the opinion was expressed that such sales were undesirable, even when all the formalities of the Pharmacy Acts were observed. So long as certain conditions are complied with no offence against the poison law is committed in selling narcotic drugs to soldiers, but an Order in Council under the Defence of the Realm Act has been made which prohibits any person "to give, sell, or supply" any "intoxicant" to any of His Majesty's Forces when on duty. The Order classes as intoxicants "any sedative, narcotic, or stimulant drug or preparation," so that a means is provided of checking to some extent a most undesirable practice. The Order does not prohibit the sale of an "intoxicant" to soldiers not on duty unless it is sold with evil intent or when the soldiers are at a port for embarkation on a ship, but if it were found that the sale of morphine and cocaine to soldiers not on duty still continues to any considerable extent it would not be surprising if the Order were made more comprehensive.

### GIFTS AND BEQUESTS.

The Stockport Infirmary will ultimately benefit to the extent of about £800, being residuary estate under the will of the late Thomas Redfern, Stockport. The infirmary has received a donation of 100 guineas from Major William Chaloner, St. Peter's Square, Stockport.

Miss Elizabeth Weir, of Edinburgh, who died on November 13 last, left £5,000 to the Royal Infirmary, Edinburgh, in memory of her brother, Mr. James Alexander Weir, of Kilbagie; and £1,000 each to the Royal Edinburgh Hospital for Incurables, and Royal Victoria Hospital for Consumption.

Mrs. Helena Schubach, of Maida Vale, London, W., who died on February 9, left £1,500 for division among various institutions, including:—King Edward's Hospital Fund for London, Charing Cross Hospital, the Jews' Hospital and Orphan Asylum and School, the Jewish Hospital for Incurables, and the London Hospital.

The annual report of the Victoria Infirmary, Helensburgh, states that the legacies intimated during the year included £100 from the late Mrs. Ure, Cairndhu; and £500 from the late Mrs. Greenlees, Moss-side. Miss MacDonald of Belmore had given a special donation of £100 in recognition of the attention paid to one of her maids while a patient in the infirmary.

Mr. James Stanley Newton Boyd, M.B., F.R.C.S., who died on February 1, left estate of the value of £32,646. After providing for legacies for some servants, he bequeathed the residue of his property in trust for his mother and sister and the survivor of them, and, subject thereto, he left two thousand guineas to Epsom College for one foundation scholar, and the ultimate residue to London University for a professorship of pathology in the Medical School of Charing Cross Hospital. Out of the property belonging to his late wife he bequeathed £1,000 each to the London School of Medicine for Women, the New Hospital for Women, and the Pathological Department of that hospital; and any residue, after a number of bequests to relatives, is to go to the New Hospital for Women.

### Buildings Contemplated.

Batley.—The Batley and District Hospital will be enlarged, at an estimated cost of £4,500.

Blackadon.—The Plymouth Lunatic Asylum Visiting Committee have approved plans for the extension of the asylum buildings at Blackadon, and have forwarded the plans to the Board of Control.

Caistor.—The Rural District Council have decided to ask the Local Government Board to sanction a loan of £1,800 for building a tuberculosis block at the Isolation Hospital.

Streetly.—The Lichfield Rural District Council have passed plans for alterations to the Longfield Auxiliary Hospital at Streetly, subject to a ventilating column being provided.

Tottenham.—An inquiry has been held by the Local Government Board into an application of the Urban District Council for permission to borrow £8,643 for the purchase of land at Devonshire Hill, White Hart Lane, as a site for an isolation hospital.

Wooler.—At a recent meeting of the Northumberland County Council it was stated that land has been purchased at Wooler as a site for a sanatorium.

Rates of Subscription: Twelve months, 6/6; Six months, 4/-; Three months, 2/-, post free to any address in the United Kingdom. Abroad, Twelve months, 8/8; Six months, 5/-; Three months, 2/6, post free.—Address The Subscription Dept., "The Hospital." The Hospital Building, 28/29 Southampton Street, Strand, London, W.C.

